# Metamaterial Absorbers for Infrared Detection of Molecular Self-Assembled Monolayers

**DOI:** 10.1038/srep12570

**Published:** 2015-07-31

**Authors:** Atsushi Ishikawa, Takuo Tanaka

**Affiliations:** 1 Department of Electrical and Electronic Engineering, Okayama University, 3-1-1 Tsushimanaka, Kitaku, Okayama, Okayama 700-8530, Japan; 2 Metamaterials Laboratory, RIKEN, 2-1 Hirosawa, Wako, Saitama 351-0198, Japan; 3 Research Institute for Electronic Science, Hokkaido University, N21W10 Kitaku, Sapporo, Hokkaido 001-0020, Japan; 4 Department of Innovative and Engineered Materials, Tokyo Institute of Technology, 4259 Nagatsutacho, Midoriku, Yokohama, Kanagawa 226-8503, Japan

## Abstract

The emerging field of plasmonic metamaterials has introduced new degree of freedom to manipulate optical field from nano to macroscopic scale, offering an attractive platform for sensing applications. So far, metamaterial sensor concepts, however, have focused on hot-spot engineering to improve the near-field enhancement, rather than fully exploiting tailored material properties. Here, we present a novel spectroscopic technique based on the metamaterial infrared (IR) absorber allowing for a low-background detection scheme as well as significant plasmonic enhancement. Specifically, we experimentally demonstrate the resonant coupling of plasmonic modes of a metamaterial absorber and IR vibrational modes of a molecular self-assembled monolayer. The metamaterial consisting of an array of Au/MgF_2_/Au structures exhibits an anomalous absorption at ~3000 cm^−1^, which spectrally overlaps with C-H stretching vibrational modes. Symmetric/asymmetric C-H stretching modes of a 16-Mercaptohexadecanoic acid monolayer are clearly observed as Fano-like anti-resonance peaks within a broad plasmonic absorption of the metamaterial. Spectral analysis using Fano line-shape fitting reveals the underlying resonant interference in plasmon-molecular coupled systems. Our metamaterial approach achieves the attomole sensitivity with a large signal-to-noise ratio in the far-field measurement, thus may open up new avenues for realizing ultrasensitive IR inspection technologies.

Light absorption, which is one of the most fundamental light-matter interactions, is an essential phenomenon in a variety of the optical applications, such as photovoltaic cells and thermal management^1^,^2^. A material with a large absorption constant may exhibit strong light absorption, but such a material, on the other hand, also shows a strong light reflection due to strong impedance mismatching at the interface. The emerging field of plasmonic metamaterials, artificial materials composed of metallodielectric nanostructures, has defied this common sense, where material resonances and dispersions can be tailored at will^3^,^4^,^5^. One important consequence of such a capability is that two macroscopic optical properties, refractive index and characteristic impedance, can be engineered independently, allowing for the ultimate control of light^6^,^7^,^8^,^9^,^10^,^11^,^12^. Recent development of metamaterial absorbers has shown to exhibit large or even perfect absorption within a certain frequency range^13^,^14^,^15^,^16^,^17^. Since the metamaterial absorber offers a unique surface condition with tailored absorption properties as well as strong plasmonic enhancement, a variety of potential applications has been proposed, such as high-efficiency thermal emitter and high-sensitive bio-chemical sensing^18^,^19^.

Infrared (IR) absorption spectroscopy of molecular vibrations is of importance in material/medical science and security detection, since it provides essential information of the molecular structure, composition, and environment. For the versatile applications in prompt/easy-to-use IR inspection technologies, direct (far-field) detection of extremely small amounts of molecules is required. Although IR absorption spectroscopy offers fairly large signal intensity for practical applications, IR detection of picomole-level specimens, e.g., a monomolecular film, is still challenging. Surface-enhanced IR absorption (SEIRA) has been extensively studied and dramatic improvements of the sensitivity by several orders of magnitude were demonstrated using tailored plasmonic nanostructures^20^,^21^,^22^,^23^.

Recent efforts to reach atto/zeptomole sensitivity of SEIRA, however, have focused on hot-spot engineering to improve the near-field enhancement and its spatial and spectral mode overlapping between the plasmons and molecular vibrations^24^,^25^,^26^. On the other hand, background suppression is an alternative approach to gain a large signal-to-noise ratio for better sensitivity^19^. Here, we propose a novel spectroscopic technique based on the metamaterial IR absorber allowing for a low-background detection scheme as well as significant plasmonic enhancement. Specifically, we experimentally demonstrate the resonant coupling of plasmonic modes of a metamaterial absorber and IR vibrational modes of a molecular self-assembled monolayer (SAM). The sensitivity is then improved based on the dark reference measurement, where the vibrational signals are detected as distinct anti-resonance peaks within a strong absorption of the metamaterial. Our metamaterial approach achieves the attomole sensitivity with a large signal-to-noise ratio in the far-field measurement, thus further lowering the detection limit of direct IR absorption spectroscopy.

Figure 1(a) shows a unit cell cross-section of a metamaterial IR absorber consisting of a Au micro-ribbon on a thick Au film separated by a MgF_2_ gap layer. The Au ribbon width, *w*, and unit cell dimension, Λ, were 1.5 μm and 3 μm, respectively. The surface structure was purposely designed to exhibit an anomalous IR absorption at ~3000 cm^−1^, which spectrally overlapped with C-H stretching vibrational modes. The fabrication process started with electron beam evaporation of a 200-nm Au film onto a SiO_2_ substrate with a 5-nm Cr adhesion layer. Using a standard photolithography process, an array of one-dimensional (1D) micro-ribbon structures was patterned on the Au film surface. The surface structure was then obtained after 30-nm MgF_2_ and 50-nm Au films deposition and liftoff process. Figure 1(b,c) show a photograph of the fabricated metamaterial sensor chip with a total area of 26 × 26 mm^2^ and its SEM image, demonstrating uniform surface structures over a large chip area.

The absorption property of the metamaterial absorber was characterized by using a Fourier-transformed infrared spectrometer (FTIR) equipped with a variable angle reflection accessory (JASCO, FT/IR-6300FV and Harrick, Seagull). Figure 1(d) shows an experimental setup of reflection measurement by changing the incident angle, θ, of the p-polarized light. To improve the signal-to-noise ratio of IR signal, a sample chamber was purged with dry nitrogen gas, and liquid nitrogen-cooled high-sensitive MCT (HgCdTe) detector was used with the frequency resolution of 2 cm^−1^. Due to the presence of a thick Au film on the substrate, there is no transmittance and an incident IR beam is either reflected or absorbed by the surface structure. Since the excitation of plasmon modes of the metamaterial manifests as absorption dips in the reflection spectrum, we have evaluated the reflection spectrum of the metamaterial normalized by that of a bare Au reference sample.

Figure 2(a) shows the measured reflectance map of the metamaterial absorber as a function of incident angle and frequency. Depending on the incident angle, three major valleys of nearly 100% absorption were clearly observed from 1000 to 5000 cm^−1^ (*f* = 30–150 THz), in addition to several minor absorptions. To identify the underlying plasmon modes for these absorptions, a set of numerical simulations was carried out using the finite-element-method (FEM). In the calculations, the refractive index of SiO_2_ was set at 1.45 and the empirical values were used for Au and MgF_2_^27^,^28^. Figure 2(b) shows the corresponding numerical result, and it well re-produced the experimental one both qualitatively and quantitatively. The minor absorptions can be attributed to the Fabry-Perot resonances of surface plasmon polaritons (SPPs) propagating on the metamaterial with the 1D periodic surface structures (Supplementary Fig. S1). Their dispersion relations roughly satisfy the momentum conservation of the SPP excitation, i.e., (*ω*/*c*)*sin*θ = (π/Λ)*l*, where *ω* is the angular frequency, *c* is the speed of light, and *l* is integer. On the other hand, the frequencies of the major absorptions, labeled by m = 1, 2, and 3, are independent of the incident angle, and they are attributed to the localized plasmon resonances of the Au/MgF_2_/Au structure.

Figure 3 shows the corresponding electromagnetic field distributions (H_y_ and E_z_) of the major absorption dips at θ = 80°. Note that all figures were vertically enlarged 3 times for clarity. For an incident p-polarized light, localized plasmons are resonantly excited in the upper Au ribbon with dipole orientation along the ribbon width. The bare localized plasmons then interact with their own mirror images in the thick Au film, and the plasmon hybridization forms two new eigenmodes, symmetric and asymmetric ones^29^. However, since the symmetric modes are naturally prohibited due to the parity of image interaction, only the asymmetric modes are selectively excited in this system. Because the asymmetric modes, so-called magnetic modes, associate with out-of-phase charge oscillation, the incident and re-radiated lights are destructively interfered and the reflected light is effectively cancelled out. Owing to this physical mechanism of the metamaterial absorber, the unwanted light reflection from the Au surface is suppressed, resulting in the strong light absorption.

Since the net electric dipole moment of such magnetic modes is zero in the quasistatic limit, the mode excitation is naturally weak and strongly depends on the retardation effect induced by the oblique incidence. In the case of m = 2 [Fig. 3(b)], since the electric filed distribution is totally symmetric across the center of the surface structure, the mode is ideally dark and cannot be excited at the normal incidence. As the incident angle increases, the mode is getting excited due to the symmetry breaking along the x axis and the absorption dip becomes pronounced at θ > 30° (Fig. 2). A similar situation is also observed for the m = 3 case; the mode becomes dark and cannot be excited at the specific incident angle of 40° where the retardation effect is cancelled out due to the asymmetric mode profile.

Since the metamaterial absorber offers not only tailored plasmonic enhancement but also significant background suppression, such a unique surface environment can provide sensitive IR detection scheme of extremely small amounts of molecules. Here, we explore low-background resonant SEIRA by utilizing the resonant coupling between the plasmonic modes of a metamaterial absorber and IR vibrational modes of a molecular SAM. 16-Mercaptohexadecanoic acid (16-MHDA, Shigma-Aldrich), shown in the inset of Fig. 4(a), was used as a target molecule to exhibit typical symmetric/asymmetric C-H stretching vibrational modes at ~2855/2920 cm^−1^. The m = 2 mode of the metamaterial, which spectrally overlapped with these vibrational modes, was employed to form a plasmon-molecular coupled system. The self-assembling process of a molecular monolayer started by immersing the metamaterial into a 16-MHDA ethanol solution with a concentration of 10^−3^ M^30^. After 48 hours, the sample was completed by washing with ethanol and drying with desiccated nitrogen gas. The metamaterial was then totally covered by a 21.5-Å thick SAM of the 16-MHDA with their thiol head-group chemisorbed on the Au surface. For the reference, a bare Au sample was also prepared using the same process.

Figure 4(a) shows the measured reflectance spectra of the 16-MHDA SAM on a bare Au surface at θ = 80° (top) and on the metamaterial for different incident angles from 30° to 70° (bottom). For the bare Au case, the spectrum naturally suffered from an extremely low signal-to-noise ratio, thus being hard to detect the respective absorption dips of the C-H stretching modes. The metamaterial, on the other hand, exhibited a broad plasmonic absorption at *ω*_pl_ = 2921.9 cm^−1^ and the dip became pronounced as the incident angle increased. With the molecules in the vicinity of the surface structure, their vibrational modes resonantly coupled with the plasmonic modes of the metamaterial. This in turn produced distinct Fano-like anti-resonant peaks within a broad absorption of the metamaterial, and this resonant coupling process depended on the incident angle. The signal intensities reached their maxima around θ = 40° while no clear vibrational signal was obtained at θ < 30° or θ > 70°. When θ < 30°, anti-resonant peaks were too weak to detect because of the weak excitation of the plasmonic mode of the metamaterial. At θ > 70°, on the other hand, the molecular vibrational modes were excited directly by the incident IR light, rather than by the resonant coupling process, and these competing processes rendered the signals weak.

The net vibrational excitation of molecules can be extracted by the baseline correction to divide the measured reflectance spectrum by the line-shape of the plasmon resonance^31^. Figure 4(b) shows the extracted vibrational signal at θ = 40° (top). The respective vibrational signals of the symmetric/asymmetric C-H stretching modes are clearly observable, demonstrating our primary goal of realizing metamaterial-enhanced IR absorption spectroscopy. To quantitatively analyze the vibrational signal, Fano line-shape fitting was also carried out with the following function form^32^. 
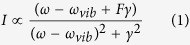
 where *ω*_vib_ is the resonant frequency, *γ* is the damping constant (HWHM), and *F* is the Fano parameter to describe the degree of asymmetry. The spectral line-shapes of the experimental result were well re-produced by the Fano fitting curves with the corresponding parameters [bottom in Fig. 4(b)]. The center frequencies of the fitting curves were in good agreement with those of the symmetric/asymmetric C-H stretching modes, making precise identification of a specific functional group practically possible. With a frequency detuning between the plasmon resonance and molecular vibrations, the fitting curve was clearly modified from a symmetric line-shape (*F* = 0) to asymmetric one (*F* = −0.05) due to the nature of the Fano resonance. Using the SAM packing density of 21.4 Å^2^/molecule, the sensitivity was estimated to be ~1.8 attomoles within the diffraction-limited IR beam spot in the FT-IR reflection measurement^33^. By optimizing the surface structure to achieve large mode overlapping between the plasmons and molecular vibrations, the sensitivity can be further improved down to zeptomole level^34^.

In conclusion, a novel spectroscopic technique based on metamaterial-enhanced IR absorption of a molecular SAM was proposed and demonstrated. The low-background detection scheme with tailored plasmonic enhancement by the metamaterial absorber achieved the attomole sensitivity of direct IR absorption spectroscopy. Spectral analysis using Fano line-shape fitting revealed the underlying resonant interference in plasmon-molecular coupled systems. Our metamaterial approach may open up new avenues for realizing ultra-sensitive IR inspection technologies.

## Additional Information

**How to cite this article**: Ishikawa, A. and Tanaka, T. Metamaterial Absorbers for Infrared Detection of Molecular Self-Assembled Monolayers. *Sci. Rep.* **5**, 12570; doi: 10.1038/srep12570 (2015).

## Supplementary Material

Supplementary Information

## Figures and Tables

**Figure 1:**
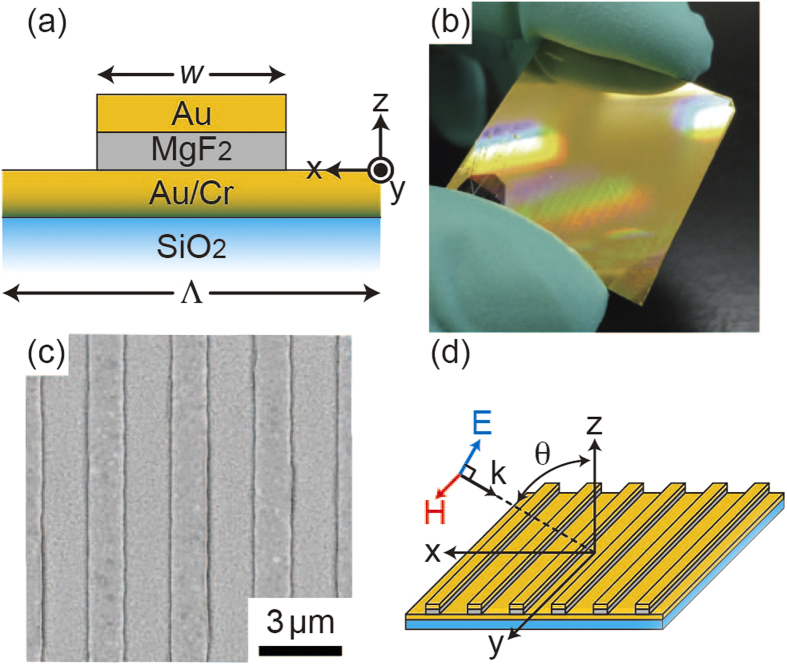
Design and fabrication of metamaterial absorber. (**a**) Schematic unit cell cross-section of a metamaterial IR absorber consisting of a 50-nm Au micro-ribbon on a thick Au film separated by a 30-nm MgF_2_ gap layer. (**b**) Photograph of the fabricated metamaterial with a total area of 26 × 26 mm^2^ and (**c**) its SEM image, revealing that the width, *w*, and unit cell dimension, Λ, were 1.5 μm and 3 μm, respectively. (**d**) Experimental setup of FT-IR reflection measurement by changing the incident angle, θ, of the p-polarized light.

**Figure 2:**
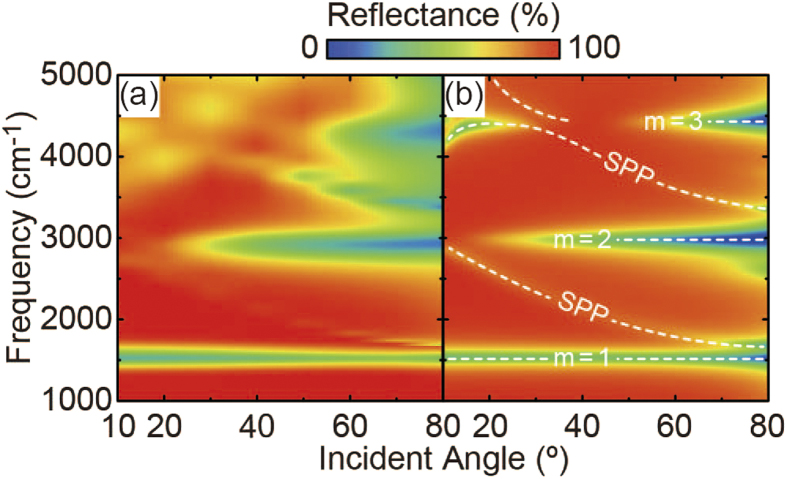
IR characterization of metamaterial absorber. (**a**) Measured reflectance map of the metamaterial absorber as a function of incident angle and frequency. (**b**) Numerically simulated reflectance map, which well re-produced the experimental result qualitatively and quantitatively. The white dotted curves in (**b**) indicate the excited plasmon modes.

**Figure 3:**
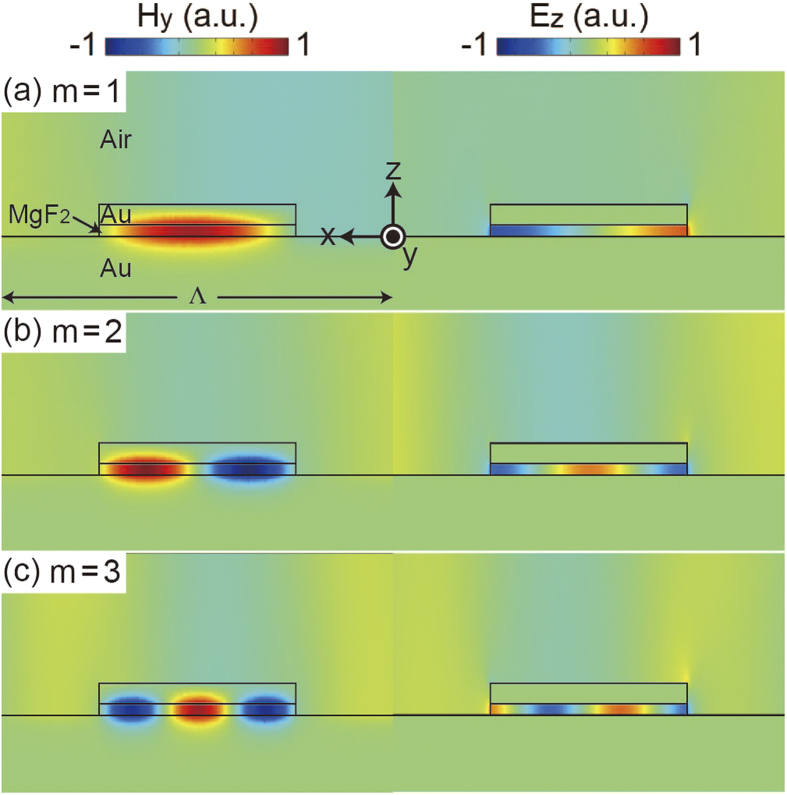
Mode profiles of metamaterial absorber. Corresponding H_y_ and E_z_ distributions of the major absorption dips at θ = 80° in Fig. 2(b): (**a**) m = 1 at 1540 cm^−1^, (**b**) m = 2 at 3013.3 cm^−1^, and (**c**) m = 3 at 4476.6 cm^−1^. All figures were vertically enlarged 3 times for clarity.

**Figure 4:**
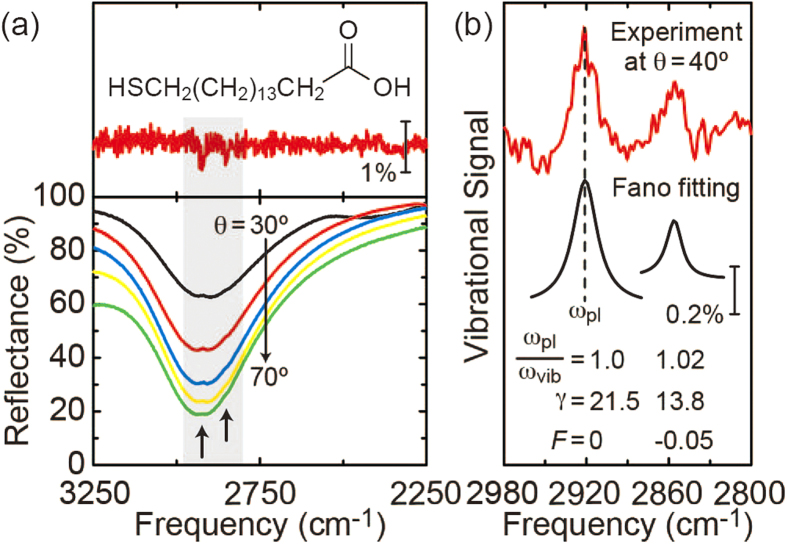
Metamaterial-enhanced IR absorption. (**a**) FT-IR reflection spectra of the 16-MHDA SAM on a bare Au surface at θ = 80° (top) and on the metamaterial absorber at θ = 30° to 70° (bottom). SAM of 16-MHDA [the inset of (**a**)] exhibited two absorption dips in the shaded region, corresponding to the symmetric/asymmetric C-H stretching vibrational modes at ~2855/2920 cm^−1^. Fano-like anti-resonant peaks, indicated by the black arrows, arose from the resonant coupling between the plasmonic mode of the metamaterial and the molecular vibrational modes. (**b**) Vibrational signal at θ = 40° extracted by the baseline correction (top) and the corresponding Fano fitting curves with the frequency detuning, *ω*_pl_/*ω*_vib_, damping constant, *γ* (cm^−1^), and Fano parameter, *F*, used in Eq. (1) (bottom).
