# Supplementary material for: Metamaterial Absorbers for Infrared Detection of Molecular Self-Assembled Monolayers
Source: Sci Rep. 2015 Jul 31;5:12570. doi: 10.1038/srep12570 (PMC4521146; doi:10.1038/srep12570)
Supplement: Supplementary Information [file srep12570-s1.pdf]

Supplementary Information

Metamaterial Absorbers for Infrared Detection of Molecular Self-Assembled Monolayers

Atsushi Ishikawa[1,2,a)] and Takuo Tanaka[2,3,4]

[1]*Department of Electrical and Electronic Engineering, Okayama University, 3-1-1 Tsushimanaka, Kitaku, Okayama, Okayama 700-8530, Japan*

[2]*Metamaterials Laboratory, RIKEN, 2-1 Hirosawa, Wako, Saitama 351-0198, Japan*

[3]*Research Institute for Electronic Science, Hokkaido University, N21W10 Kitaku, Sapporo, Hokkaido 001-0020, Japan*

[4]*Department of Innovative and Engineered Materials, Tokyo Institute of Technology, 4259 Nagatsutacho, Midoriku, Yokohama, Kanagawa 226-8503, Japan*

[a)] Electronic mail: a-ishikawa@okayama-u.ac.jp.

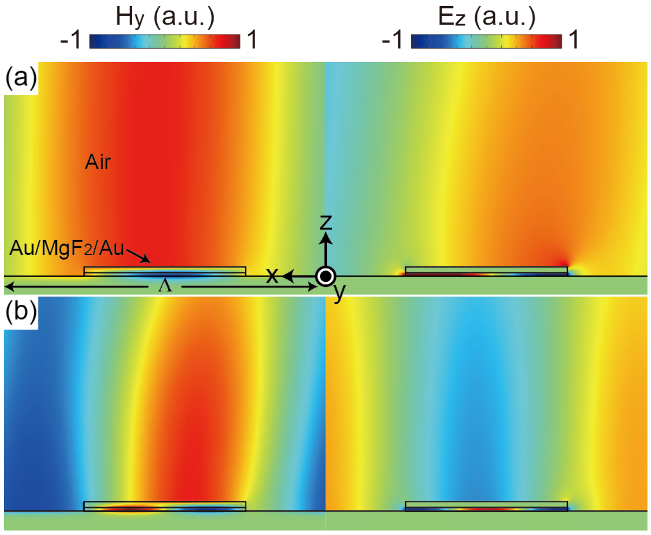

Figure S1 | Mode profiles of SPPs. Corresponding H_y and E_z distributions of the minor absorption dips at $\theta = 80^o$ in Fig. 2(b): (a) at 1690 cm^{-1} and (b) at 3370 cm^{-1}.
